# The Effect of Discharge Planning Videos and Booklets on Quality of Life Among Patients With Heart Failure: Quasi-Experimental Study

**DOI:** 10.2196/75417

**Published:** 2025-09-05

**Authors:** Fitri Arofiati, Fuji Dwi Lestari, Henri Setiawan

**Affiliations:** 1 Department of Nursing, Universitas Muhammadiyah Yogyakarta, Jl. Brawijaya, Kasihan, Bantul, 55183, Indonesia, 62 81392462014; 2 Department of Nursing, STIKes Muhammadiyah Ciamis, Ciamis, Indonesia

**Keywords:** discharge planning, heart failure, patient-centered care, quality of life, video

## Abstract

**Background:**

Heart failure remains a major global health issue, significantly impacting patients’ quality of life due to its chronic and progressive nature. Effective discharge planning, including educational interventions such as videos and booklets, plays a crucial role in enhancing self-care management and overall patient well-being.

**Objective:**

The aim of this study is to evaluate the effects of discharge planning videos and booklets on the quality of life of patients with heart failure.

**Methods:**

This study used a quasi-experimental design and was conducted at PKU Muhammadiyah Gamping Hospital from July to November 2024. A total of 42 participants who met the inclusion criteria were selected based on sample size calculations using G*Power and were evenly assigned to intervention and control groups. Both groups received standard discharge planning provided by health care professionals. Discharge planning videos and booklets were developed as educational tools for the intervention group. The Minnesota Living With Heart Failure Questionnaire was used to assess quality of life. The independent sample *t* test was used to analyze the effect of the intervention using SPSS (version 29). This study was conducted in accordance with the ethical principles outlined in the Declaration of Helsinki and was approved by the institutional review board (number 150/KEP-PKU/VII/2024).

**Results:**

The intervention significantly improved the quality of life of patients with heart failure, with the mean score decreasing from 39.00 (SD 8.11) to 24.76 (SD 4.02; *P*<.001) in the intervention group. In contrast, the control group showed minimal change, from 39.90 (SD 5.89) to 40.24 (SD 5.84), resulting in a statistically significant between-group difference of 15.58 (*P*<.001). Furthermore, the effect size was large (Cohen *d*=3.09), suggesting a strong practical significance of the intervention in enhancing the quality of life among patients with heart failure. Moreover**,** the mean Minnesota Living With Heart Failure Questionnaire scores across 4 domains—physical, mental, emotional, and social—also showed significant improvements after the intervention. The intervention group experienced reductions in all domains: physical (9.95 to 6.76), mental (7.81 to 5.62), emotional (13.19 to 7.48), and social (8.05 to 4.90), whereas the control group showed minimal or no change. These results indicate that the intervention effectively improved patients’ quality of life across multiple dimensions.

**Conclusions:**

Discharge planning through videos and booklets may improve the quality of life of patients with heart failure compared to standard care. These findings highlight the potential clinical value of structured patient education. The intervention appeared to enhance patients’ understanding of their condition and support self-management behaviors, including adherence to lifestyle recommendations. However, they should be interpreted with caution and confirmed through further studies with larger and more diverse populations.

## Introduction

Heart failure is a chronic and progressive condition that significantly impairs the quality of life (QoL) of patients globally. Characterized by the heart’s inability to pump blood efficiently, it manifests through symptoms such as shortness of breath, fatigue, and fluid retention. Despite advancements in medical treatments, heart failure remains a leading cause of hospitalization and mortality, imposing a substantial burden on health care systems and patients alike [1-3]. In Indonesia, heart failure is among the top 10 noncommunicable diseases, with 229,696 (0.13%) patients diagnosed with the condition. Additionally, based on doctor’s diagnoses or symptoms, the prevalence of heart failure in Indonesia is estimated at 1.5%, affecting approximately 2,650,340 individuals [4]. These alarming statistics highlight the urgent need for effective management strategies to mitigate the growing burden of heart failure.

The management of heart failure is multifaceted, requiring not only pharmacological interventions but also comprehensive patient education and self-care strategies [5,6]. Unhealthy lifestyles and the inability of patients to independently manage their condition are significant contributors to the rising incidence of heart failure. Addressing these factors necessitates a focus on enhancing self-management among patients with heart failure. In this context, discharge planning emerges as a critical component in ensuring that patients are equipped with the necessary knowledge and skills to manage their condition effectively after leaving the hospital [7,8].

Discharge planning is a multidisciplinary process designed to facilitate the transition of patients from hospital to home, thereby reducing the risk of readmission and improving overall health outcomes. It involves the coordination of care, patient education, and the provision of resources to support self-management [7]. Numerous studies have underscored the importance of discharge planning in improving health outcomes for patients with heart failure. For instance, Rice et al [9] demonstrated that comprehensive discharge planning and postdischarge support significantly enhance health outcomes, potentially improving patients’ QoL through better education and resources [9]. Similarly, Graupner et al [10] found that structured discharge planning interventions led to improved outcomes, including enhanced QoL, increased knowledge related to heart failure, improved self-care behaviors, and reduced readmission rates. These findings underscore the critical role of well-designed discharge planning programs in addressing the multifaceted challenges faced by patients with heart failure.

Patient education is a cornerstone of effective heart failure management. Empowering patients with the knowledge and skills to actively participate in their care can lead to better adherence to treatment regimens, improved symptom management, and reduced hospital readmissions [11-13]. However, many patients face challenges such as low health literacy, cognitive impairments, or language barriers, which can hinder their ability to understand and apply the information provided. Additionally, the emotional and psychological burden of living with a chronic condition like heart failure can further complicate the educational process [14,15].

In recent years, the advent of digital technology has opened new avenues for enhancing patient education and engagement [16]. Multimedia tools, such as videos, offer a promising solution by presenting information in a clear, concise, and visually appealing manner. Videos can be tailored to address the specific needs and preferences of individual patients, making the content more relevant and effective [17-19]. For example, videos can demonstrate proper techniques for monitoring blood pressure, taking medications, or performing physical exercises, providing patients with practical guidance that they can easily follow at home. Moreover, videos can be accessed repeatedly, allowing patients to review the information as needed, which reinforces learning and promotes long-term retention [20-22].

Although videos offer a dynamic and engaging medium for patient education, booklets remain a valuable complementary tool. Booklets provide a written reference that patients can consult at their own pace, offering detailed information on various aspects of heart failure management, such as dietary recommendations, medication schedules, and warning signs of worsening symptoms [23,24]. They can also include diagrams, charts, and checklists to facilitate their understanding and application of the information. Furthermore, booklets can be customized to reflect the cultural and linguistic diversity of the patient population, ensuring that the content is accessible and relevant to all [25-27]. The integration of videos and booklets in discharge planning may offer a synergistic effect, enhancing the overall quality of patient education. Videos can capture the patient’s attention and convey key messages in an engaging manner, while booklets provide a comprehensive resource for additional details and clarification [28,29]. Together, these tools address the cognitive, emotional, and practical aspects of patient education, promoting a more holistic approach to self-care. Therefore, this study aims to evaluate the effects of discharge planning videos and booklets on the QoL of patients with heart failure. It is hypothesized that patients who receive discharge planning with video and booklet support will experience a significantly greater improvement in QoL compared to those who receive standard discharge planning alone.

## Methods

### Design and Setting

This quasi-experimental study was conducted at PKU Muhammadiyah Gamping Hospital from July to November 2024. Such studies offer a valuable alternative for estimating causal relationships and are increasingly used as more observational data become available [30].

### Participants and Sampling

The study population consisted of patients with heart failure hospitalized at PKU Muhammadiyah Gamping Hospital. The sample size was calculated using G*Power (version 3.1), applying a *t* test for independent means (two groups) with a significance level of 0.05, statistical power of 0.80, and an effect size of 0.80 (large). A large effect size (Cohen *d*=0.8) was assumed based on prior studies demonstrating marked improvements in the QoL of patients with heart failure following structured educational interventions [31,32]. A total of 42 respondents were recruited and equally divided into two groups: 21 in the intervention group and 21 in the control group. Inclusion criteria included patients who had heart failure classified as grade 1 or 2, were aged over 20 years, were literate, and were smartphone users. Exclusion criteria included patients who died, were readmitted within a month, or had more than 3 comorbidities.

### Intervention

Both groups received standard discharge planning provided by health care professionals. In addition, the intervention group received supplementary educational materials in the form of a video and a booklet, specifically developed to enhance patients’ understanding of self-care following hospital discharge. The content of these materials was guided by the Self-Care of Heart Failure Index framework and emphasized 3 core components: symptom monitoring, adherence to treatment, and self-care management.

The educational video, lasting 4 minutes and 27 seconds, included information on the definition and symptoms of heart failure, contributing risk factors, dietary recommendations, medication adherence, and strategies for home-based care. The video was uploaded to YouTube to ensure ease of access and could be rewatched as needed by patients or their caregivers [33]. The accompanying booklet (Multimedia Appendix 1) served as a written reference that reinforced the video content and included illustrations and simple language tailored to patients with varying literacy levels.

Both the video and booklet were developed collaboratively by a team of nursing lecturers, cardiologists, and cardiology ward nurses to ensure clinical accuracy and contextual relevance. The materials were reviewed through an expert validation process involving 2 cardiologists and 3 senior nurses using a structured content validity checklist. Additionally, the materials were pilot-tested with a group of 5 patients with heart failure to assess clarity, usability, and acceptability. Feedback from the pilot testing was used to refine the wording, visuals, and delivery method of the materials before full implementation in the study.

### Outcome Measurement: QoL

The Minnesota Living With Heart Failure Questionnaire (MLHFQ) was used to assess QoL across 4 domains: physical, emotional, mental, and social. The Indonesian version of the MLHFQ consists of 20 validated items (excluding question 10 due to low item correlation), scored using a 4-point Likert scale. Lower scores indicate better QoL, whereas higher scores reflect poorer perceived health status. Total scores range from 24 to 80, categorized as <24 (good), 24‐45 (moderate), and >45 (poor). The instrument showed high reliability (Cronbach α=0.954) [34,35].

### Data Collection and Analysis

Data collection occurred in 2 phases: a pretest before intervention and a posttest 4 weeks after intervention. Patients completed the MLHFQ questionnaire at both time points to assess changes in QoL (Multimedia Appendix 2). All data were analyzed using SPSS (version 29; IBM Corp). Descriptive statistical analysis was conducted for sociodemographic variables, including age, sex, education, and occupation. These characteristics were presented using frequencies and percentages for each group. The Shapiro-Wilk test was also used to assess the homogeneity of sociodemographic data distributions between groups. The Shapiro-Wilk test confirmed normal distribution (), allowing for parametric tests. Paired *t* tests were used to compare pretest and posttest scores within groups, while independent *t* tests compared differences between groups at each time point.

### Ethical Considerations

This study was conducted in accordance with the ethical principles outlined in the Declaration of Helsinki and was approved by the institutional review board of PKU Muhammadiyah Gamping Hospital, Indonesia (150/KEP-PKU/VII/2024). All participants provided written informed consent prior to participation, which included consent for the use of their data in secondary analyses. The confidentiality and privacy of participants were protected by using anonymized codes in all data records, and no personally identifiable information was collected or reported. Participants did not receive any monetary or material compensation for their involvement in the study. Additionally, no images or materials containing identifiable features of individual participants are included in this manuscript or its supplementary files.

## Results

### Sociodemographics of the Participants

**Figure 1.**
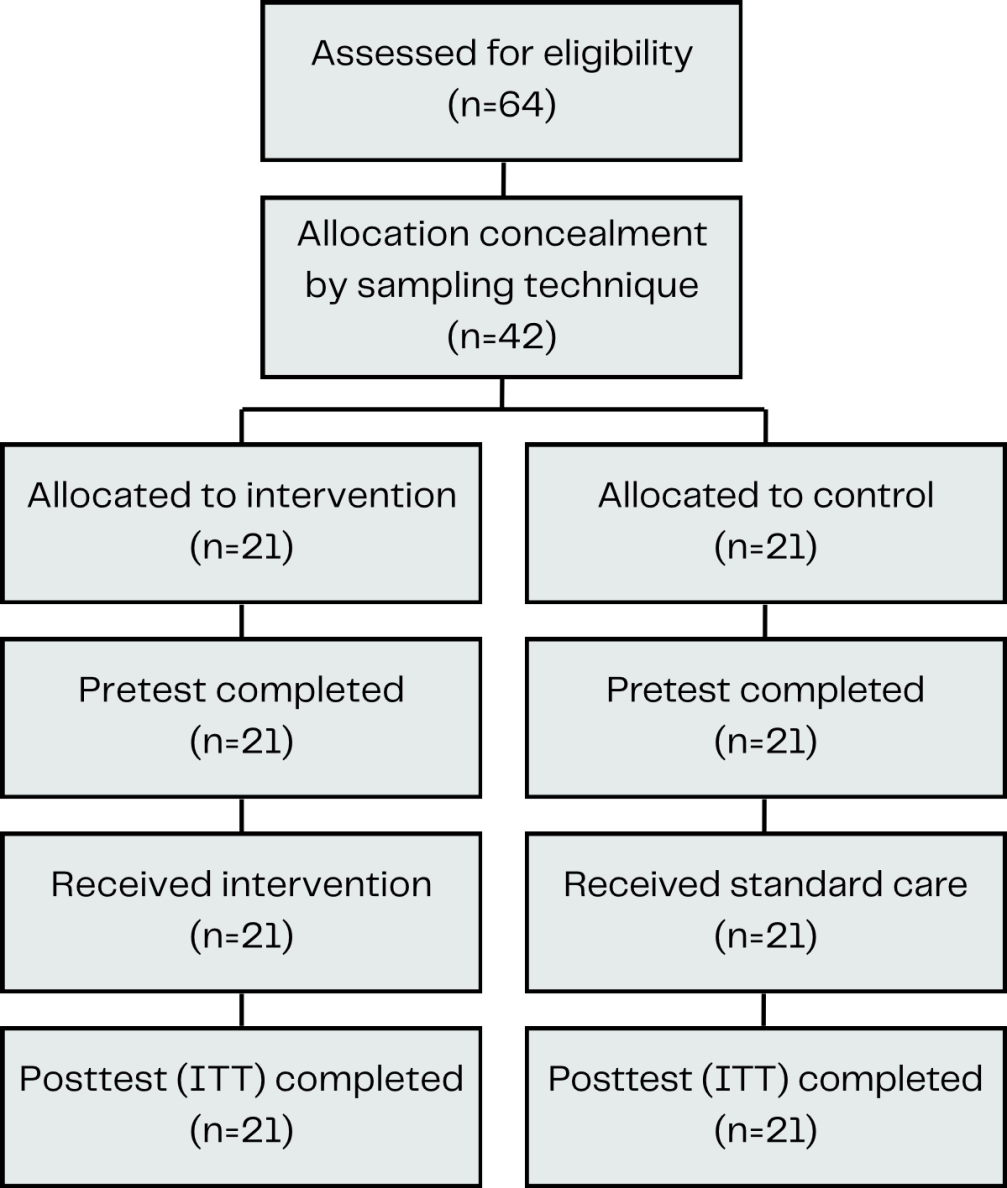
Flow diagram of participant attrition. ITT: intention-to-treat.

Detailed information on participant flow and allocation can be seen in Figure 1. Table 1 summarizes the sociodemographic characteristics of participants (n=42) in both groups. The mean age was comparable (intervention: 62.8, SD 12.3; control: 60.7, SD 15.7 y), with a higher proportion of males in both groups. Primary education was most common, and more participants in the intervention group were employed. All *P* values were >.05, indicating no significant baseline differences between groups, suggesting balanced sociodemographic characteristics.

**Table 1.** Sociodemographics of the participants.

Variables		Group		*P* value^a^
		Intervention	Control	
Age, mean (SD)		62.8 (12.3)	60.7 (15.7)	.28
Age, n (%)				
	<Mean	9 (42.9)	10 (47.6)	
	>Mean	12 (57.1)	11 (52.4)	
Sex, n (%)				.84
	Male	15 (71.4)	12 (57.1)	
	Female	6 (28.6)	9 (42.9)	
Education, n (%)				.67
	Primary	5 (23.8)	10 (47.6)	
	Secondary	2 (9.5)	2 (9.5)	
	Tertiary	10 (47.6)	8 (38.1)	
	University	4 (19)	1 (4.8)	
Occupation, n (%)				.31
	Employed	13 (61.9)	10 (47.6)	
	Unemployed	8 (38.1)	11 (52.4)	

a Homogenity test by Shapiro-Wilk.

### The Effect of the Intervention on the QoL of Patients With Heart Failure

Table 2 presents the impact of discharge planning using videos and booklets on the QoL of patients with heart failure. Before the intervention, the mean scores were similar between groups (intervention: 39.00, SD 8.11; control: 39.90, SD 5.89). After the intervention, the intervention group’s mean score significantly decreased to 24.76 (SD 4.02; *P*<.001), while the control group showed minimal change (40.24, SD 5.84; *P*=.031). The postintervention difference between groups was statistically significant (mean difference=15.58; *P*<.001), indicating that the intervention substantially improved patients’ QoL compared to standard discharge planning. Furthermore, the effect size was large (Cohen *d*=3.09), suggesting a strong practical significance of the intervention in enhancing QoL among patients with heart failure.

**Table 2.** The effect of the intervention on the quality of life of patients with heart failure.

Parameters	Group	
	Intervention	Control
Pretest, mean (SD)	39.00 (8.11)	39.90 (5.89)
Posttest^a^, mean (SD)	24.76 (4.02)	40.24 (5.84)
Significance^b^	<.001	.03

a Posttest, the mean difference between the intervention and control group was 15.58 (independent sample *t* test *P*<.001; Cohen *d*=3.09).

b Paired *t* test.

**Figure 2.**
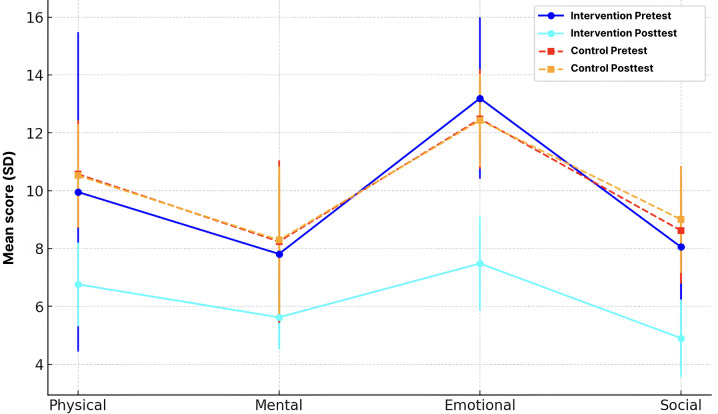
Comparison of mean scores and standard deviation between two groups.

Figure 2 illustrates the mean MLHFQ scores across 4 domains—physical, mental, emotional, and social—before and after the intervention. The intervention group showed significant reductions in all domains: physical (9.95 to 6.76), mental (7.81 to 5.62), emotional (13.19 to 7.48), and social (8.05 to 4.90). In contrast, the control group showed minimal or no change. These results indicate that the intervention effectively improved patients’ QoL across multiple dimensions.

## Discussion

### Principal Findings

The results of this study demonstrated a statistically significant improvement in the QoL of patients with heart failure who received discharge planning interventions involving educational videos and booklets, as evidenced by the independent sample *t* test (*P*<.001). Although the control group exhibited a statistically significant change, the numerical difference was minimal and is unlikely to represent a clinically meaningful effect. This change may reflect random sampling variability rather than a true treatment-related outcome. This significant difference underscores the effectiveness of these multimedia tools in enhancing patients’ understanding of self-care, promoting adherence to management strategies, and ultimately improving their overall QoL compared to the control group. The findings suggest that integrating videos and booklets into discharge planning can serve as a valuable approach in clinical settings, offering a practical and accessible means to empower patients and address the multifaceted challenges of heart failure management [36,37]. These results align with previous research emphasizing the importance of structured patient education and support in improving health outcomes, further validating the potential of such interventions to reduce the burden of heart failure and enhance patients’ well-being [38,39].

### Multimedia Tools in Patient Education

The use of educational videos in discharge planning likely contributed to the observed improvements by presenting complex medical information in a clear, engaging, and visually appealing manner. Murphy et al [40] emphasized that the dynamic nature of videos allows for the inclusion of visual and auditory elements, which cater to different learning styles, making the information more accessible to a broader audience. Similarly, Saluky and Bahiyah [41] highlighted that the ability to revisit video content allows patients to reinforce comprehension, address areas of difficulty, and improve long-term retention of key information. This aligns with studies highlighting the advantages of multimedia tools in overcoming barriers such as low health literacy, cognitive impairments, and language difficulties, which are common among patients with heart failure. By addressing these barriers, videos can enhance patients’ confidence and ability to manage their condition independently, ultimately leading to better health outcomes [42,43].

The inclusion of booklets in the intervention provided a complementary resource that allowed patients to access detailed written information at their convenience. Booklets serve as a reliable reference for patients, offering step-by-step guidance on dietary recommendations, exercise routines, and medication management [44-46]. This dual approach—combining the dynamic nature of videos with the comprehensive detail of booklets—likely created a synergistic effect, enhancing the overall impact of the discharge planning intervention. The combination of these tools addresses both the immediate and long-term educational needs of patients, providing them with the resources necessary to manage their condition effectively over time [47-50].

### Psychosocial Benefits of Multimedia-Based Discharge Planning

The significant improvement in QoL observed in this study also highlights the importance of addressing the emotional and psychological aspects of living with heart failure. Tsabedze et al [51] stated that depression and anxiety symptoms were found in over half of patients attending the congestive heart failure clinic, highlighting how chronic conditions like heart failure often lead to emotional distress and a sense of helplessness, which can hinder patients’ ability to engage in self-care [52]. By providing clear, actionable information through videos and booklets, the intervention may have alleviated some of these emotional burdens, empowering patients to take control of their health. This is particularly important given the strong link between psychological well-being and adherence to treatment regimens. The positive outcomes observed in this study suggest that multimedia-based discharge planning can play a crucial role in fostering a sense of empowerment and resilience among patients with heart failure [53,54].

### Study Implications

The findings of this study have important implications for health care systems, particularly in resource-limited settings. Heart failure is a global health challenge that places a significant burden on health care infrastructure, with high rates of hospitalization and readmission [55,56]. Effective discharge planning interventions, such as the use of videos and booklets, offer a cost-effective and scalable solution to improve patient outcomes and reduce health care costs [57-60]. These tools can be easily disseminated and adapted to meet the needs of diverse patient populations, making them a viable option for widespread implementation. Additionally, the use of multimedia tools aligns with the growing trend of digital health solutions, which have the potential to revolutionize patient education and self-management. By leveraging technology, health care providers can deliver high-quality education to patients in a format that is both accessible and engaging [16,61].

### Strengths and Limitations

One of the key strengths of this study lies in its innovative approach to discharge planning, which combines educational videos and booklets to address the diverse learning needs of patients with heart failure. Additionally, the study adopts a holistic perspective, focusing not only on clinical outcomes but also on the emotional and psychological challenges faced by patients. This patient-centered design empowers individuals with knowledge and practical tools, promoting better self-care and overall QoL. The intervention’s scalability and accessibility further strengthen its potential, as videos and booklets are cost-effective and can be adapted to various health care settings, including resource-limited environments.

However, this study has several limitations that should be considered. First, the quasi-experimental design, while practical for real-world settings, lacks the methodological rigor of a randomized controlled trial, which limits the strength of causal inferences. Second, the short follow-up period of 4 weeks restricts the ability to assess the long-term sustainability of improvements in QoL and self-care. Third, although the intervention was designed to be scalable, its implementation in resource-limited settings may face challenges related to limited technological access, funding constraints, and staff capacity. Fourth, the study did not account for several external variables such as socioeconomic status, family support, and comorbidities, which could have influenced patient outcomes and introduced potential confounding. Fifth, although baseline measures between groups were relatively similar, the analysis did not statistically adjust for baseline differences, which may limit the precision of the estimated intervention effects, particularly given the small sample size.

### Recommendations and Future Work

Given the positive outcomes observed, future studies should explore the long-term effects of discharge education using multimedia and printed materials, particularly in diverse health care settings and among patients with varying levels of health literacy. It is also recommended to conduct randomized controlled trials to strengthen causal inferences and examine the cost-effectiveness of such interventions. Additionally, integrating digital tools such as mobile health apps could be considered to further support patient self-management beyond hospital discharge.

### Conclusions

This study suggests that discharge planning incorporating videos and booklets may help improve the QoL of patients with heart failure compared to standard care. The intervention appeared to enhance patients’ understanding of their condition and support self-management behaviors, including adherence to lifestyle recommendations. Although the results indicate the potential value of structured, multimedia-based patient education, these findings should be interpreted with caution and considered preliminary. Further research with larger, more diverse populations is recommended to confirm the observed effects and assess broader clinical applicability.

## Supplementary material

10.2196/75417 Multimedia Appendix 1 Booklet of discharge planning.

10.2196/75417 Multimedia Appendix 2 Minnesota Living With Heart Failure Questionnaire.

10.2196/75417 Multimedia Appendix 3 Normality test results.

## Abbreviations

MLHFQ: Minnesota Living With Heart Failure Questionnaire
QoL: quality of life
