# Supplementary material for: The Effect of Discharge Planning Videos and Booklets on Quality of Life Among Patients With Heart Failure: Quasi-Experimental Study
Source: JMIR Cardio. 2025 Sep 5;9:e75417. doi: 10.2196/75417 (PMC12413017; doi:10.2196/75417)

PERAWATAN PASIEN

GAGAL JANTUNG

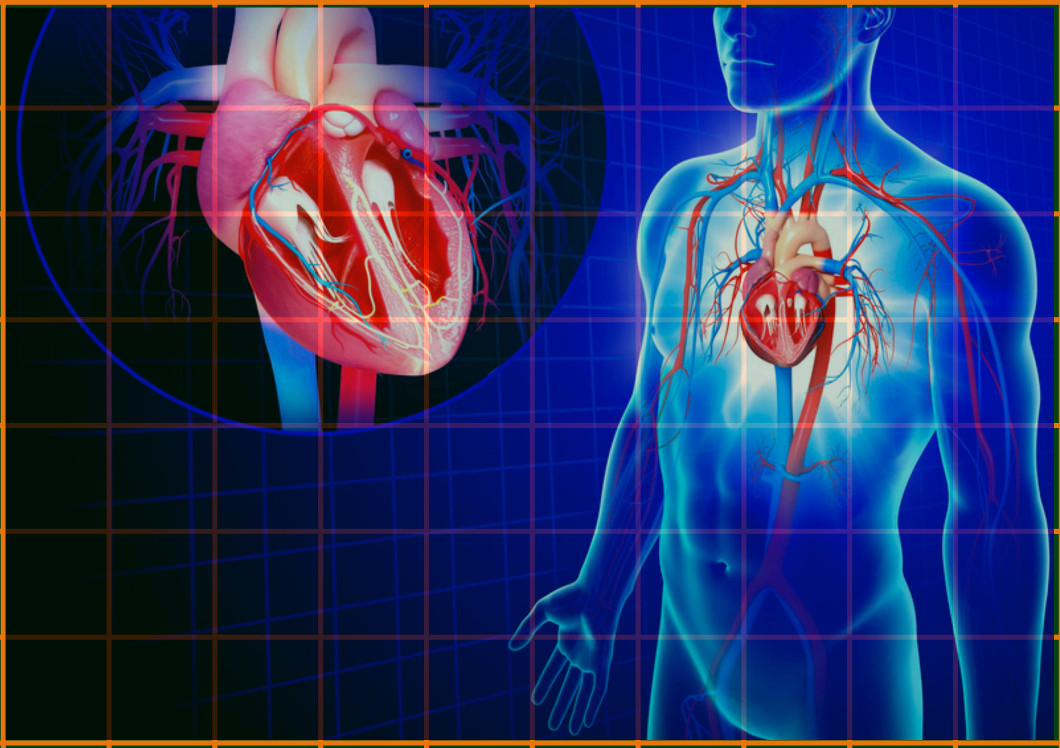

Penyusun :
Fuji DwiLestari S. Kep., Ns
Fitri Arofiati, S. Kep., Ns., MAN., Ph.D.

PRODI MAGISTER KEPERAWATAN
UNIVERSITAS MUHAMMADIYAH YOGYAKARTA

mkep.umy.ac.id mkep_umy

DAFTAR ISI :

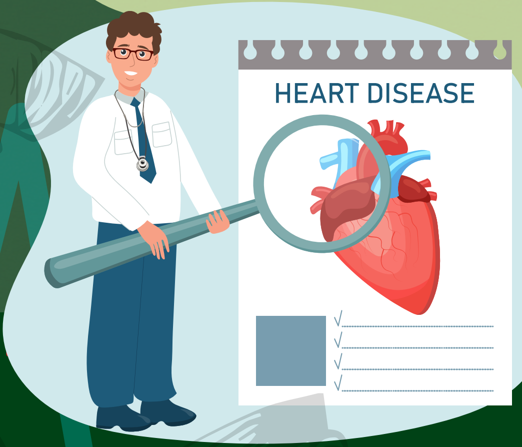

PENDAHULUAN 2

SEKILAS TENTANG GAGAL JANTUNG 3

1. Pengertian gagal jantung 4
2. Tanda dan gejala 5
3. Penyebab gagal jantung 6
4. Perawatan pasien di rumah 7
5. Perawatan pasien gagal jantung 8

MAKANAN DAN NUTRI PASIEN JANTUNG 9

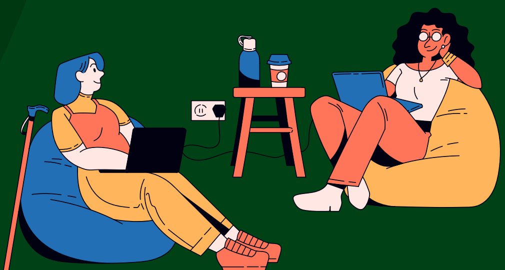

PENDAHULUAN

MASALAH KESEHATAN YANG SERING MUNCUL PADA PASIEN GAGAL JANTUNG

1. Merasa sesak nafas Saat aktifitas atau saat berbaring
2. Terjadi Pembengkakan pada tubuh seperti di pergelangan kaki, dan jantung berdebar.
3. Cepat lelah setelah berolah olahraga atau melakukan aktivitas berlebih.
4. Nafsu makan berkurang
5. Lebih sering buang air kecil dimalam hari.
6. Batuk-batuk yang tidak kunjung membaik dirasakan memberat di malam hari.
7. Sulit fokus dan konsentrasi.

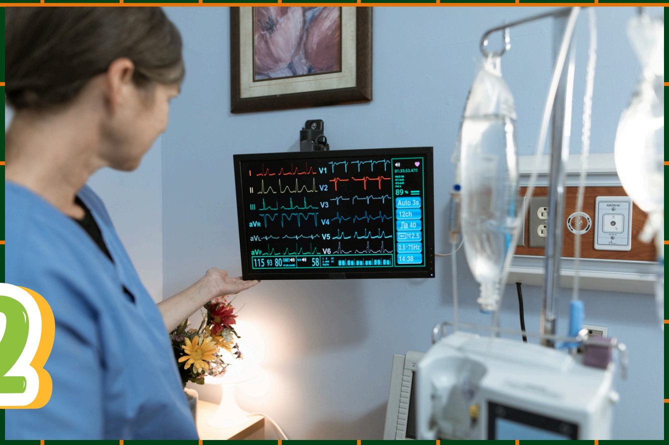

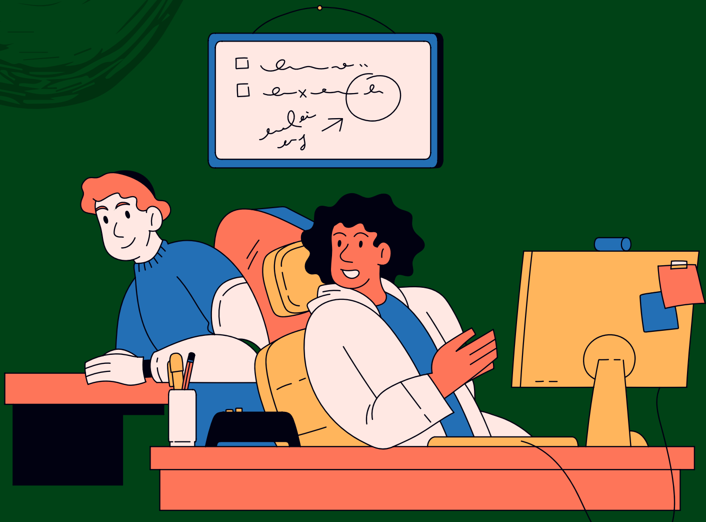

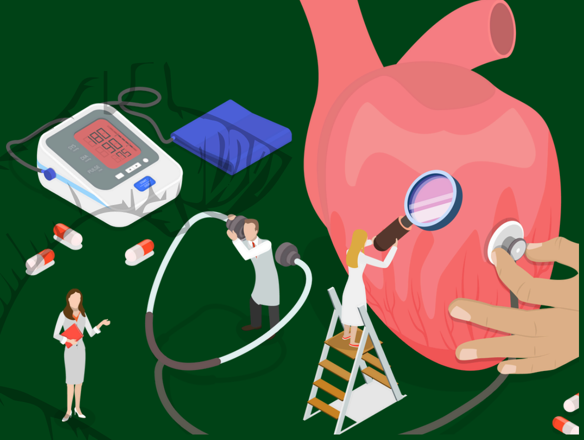

SEKILAS TENTANG GAGAL JANTUNG

KENALI FAKTOR RESIKO SERTA GEJALA DAN
"TANGANI SEGERA"

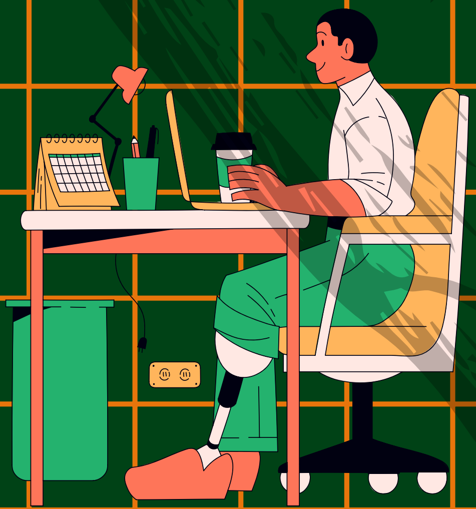

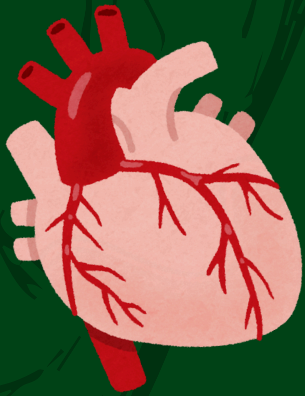

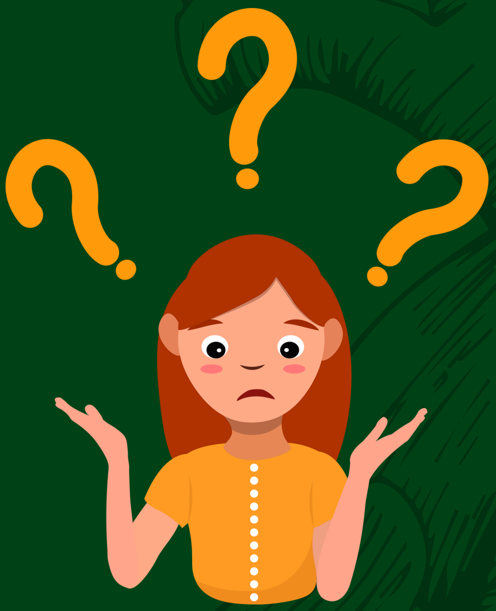

APA ITU GAGAL JANTUNG ???

Gagal jantung merupakan suatu kondisi dimana jantung tidak mampu memompa darah secara efektif karena adanya kelainan pada fungsi jantung

Gagal jantung juga merupakan suatu kondisi dimana jantung tidak mampu memompa cukup darah untuk memenuhi kebutuhan tubuh

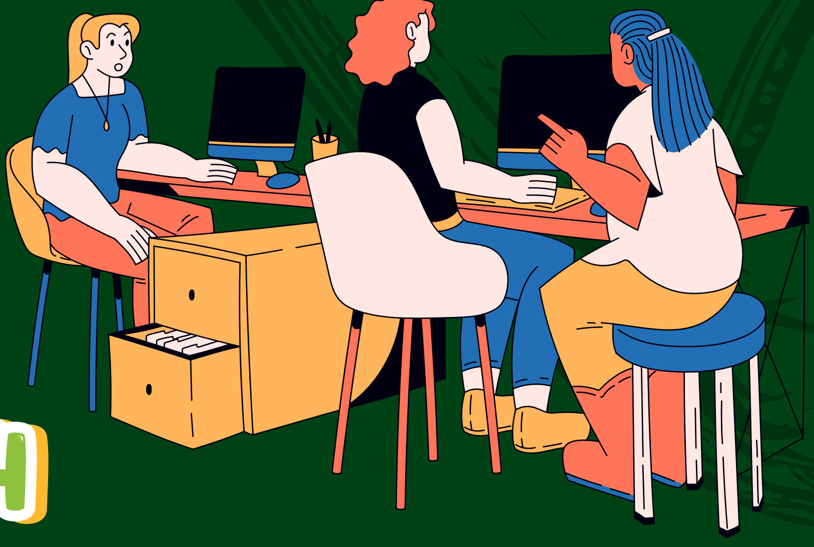

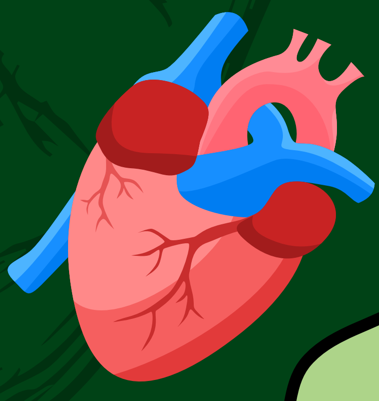

TANDA DAN GEJALA GAGAL JANTUNG

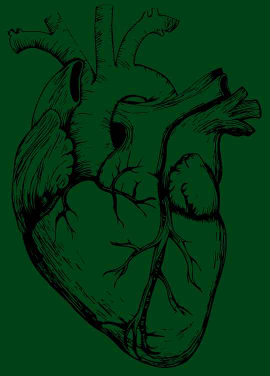

Tanda dan gejala pada gagal jantung yang mungkin bisa terjadi yaitu:

a. Kelelahan yang berlebih.
b. Keterbatasan aktivitas
c. Meningkatnya berat badan
d. Sesak nafas saat beraktivitas, pucat dan sakit kepala
e. Bengkak pada kaki

PENYEBAB GAGAL JANTUNG

1. Berkurangnya darah yang masuk keparu-paru, kelainan katub jantung, kelainan otot jantung.
2. Terjadinya peningkatan darah (Darah Tinggi)
3. Adanya Riwayat asma atau sesak nafas.
4. Terdapat infeksi
5. Konsumsi garam dan cairan berlebih .

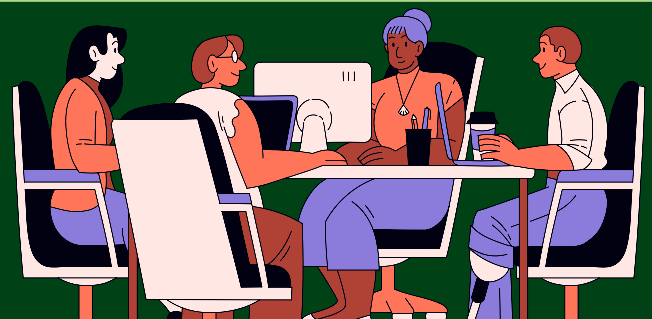

PERAWATAN PASIEN DI RUMAH

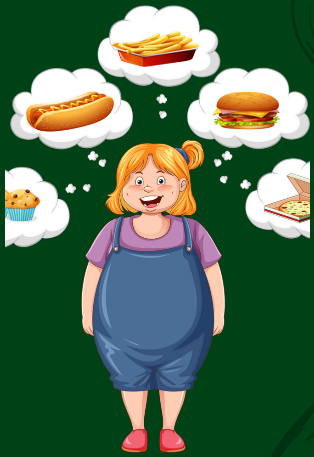

Mengatur Makanan yang di konsumsi dan menurunkan Berat Badan pada orang gemuk (obesitas)

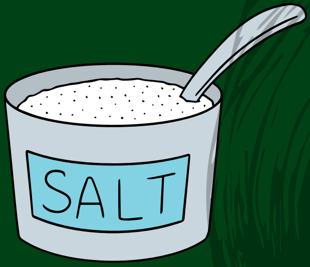

membatasi konsumsi garam berlebihan

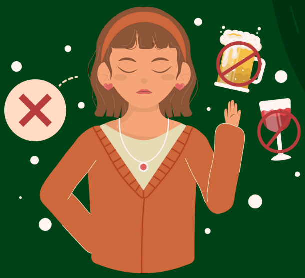

menghindari minumam beralkohol

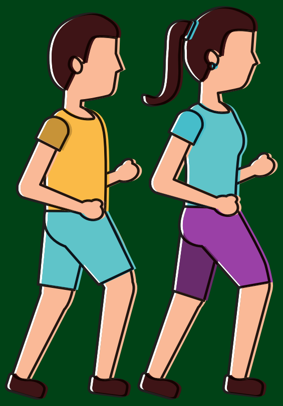

melakukan olahraga ringan untuk menguatkan otot

PERTOLONGAN PERTAMA PASIEN DIRUMAH

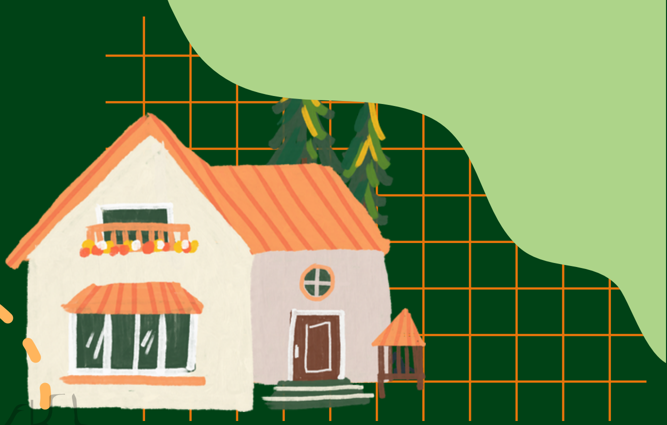

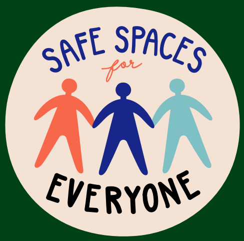

Bawa pasien ketempat yang aman

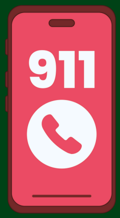

Segera hubungi 119 atau Rumah Sakit terdekat untuk pertolongan tenaga kesehatan dan ambulan

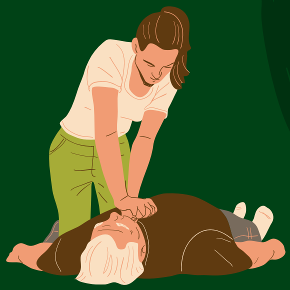

Lakukan resusitasi jantung oleh tenaga medis atau orang yang sudah terlatih

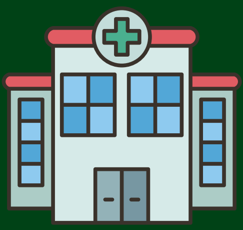

Segera bawa ke Rumah Sakit

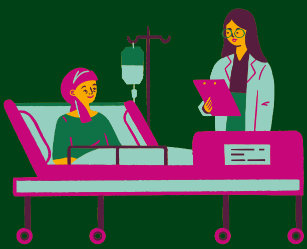

Melakukan perawatan lanjutan di Rumah Sakit

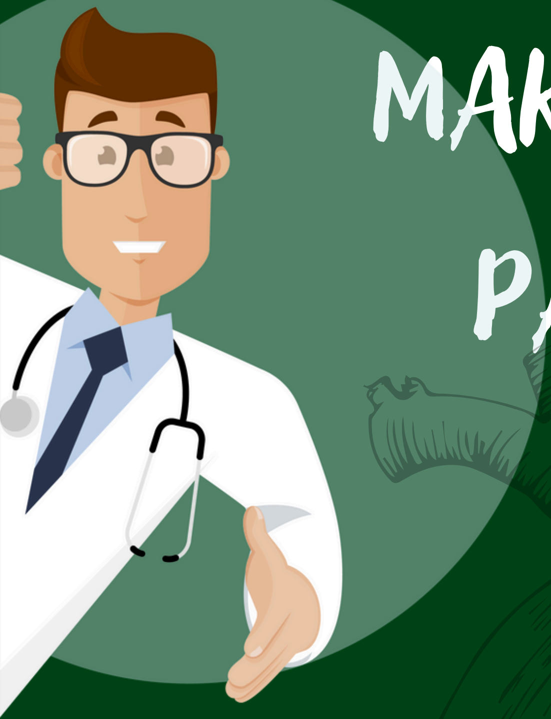

MAKANAN DAN NUTRISI PASIEN JANTUNG

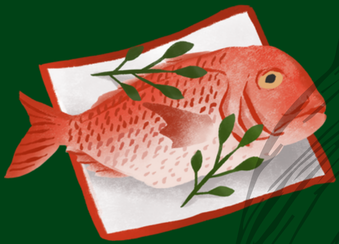

Ikan berlemak seperti Ikan kembung, Sarden, tuna dan mengandung asam lemak OMEGA 3 yang emiliki sifat anti infamasi yang baik untuk jantung

Tahu dan tempe
jika di konsumsi 1 minggu sekali bisa mencegah terjadinya penyakit jantung

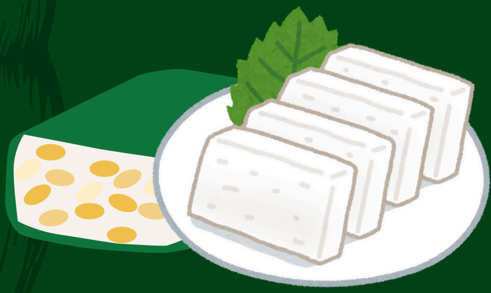

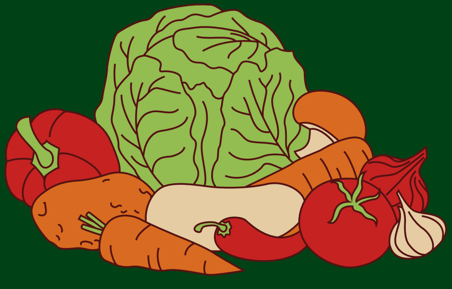

Brokoli, bayam, kangkung, sawi hijau, pak choy, dan Kentang yang kaya akan serat dan anti oksidan yang baik untuk jantung

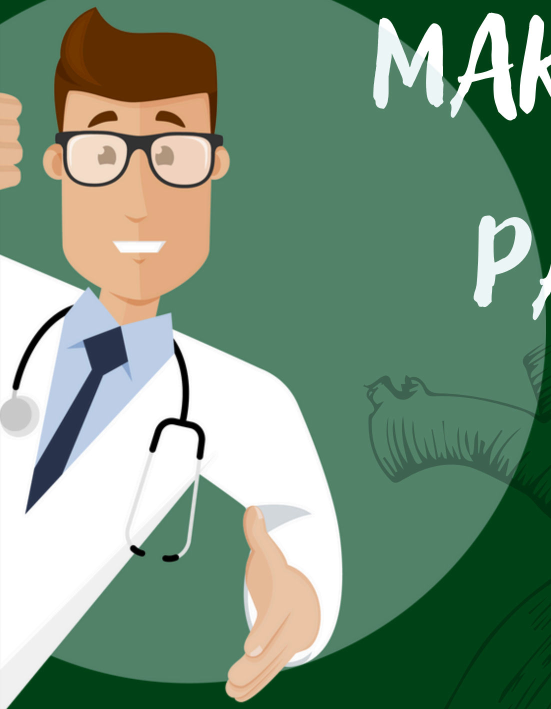

MAKANAN DAN NUTRISI PASIEN JANTUNG

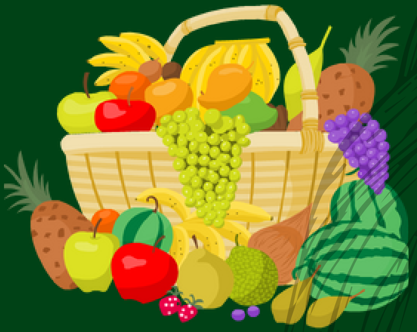

Buah Jeruk, Melon, Pepaya, Apel, buah Bit, Tomat dan Alpukat sebagai antioksidan untuk melindungi jantung

Kacang mete, kacang hitam, kacang merah, dan Biji Labu merupakan nutrisi yang mengandung serta yang tinggi

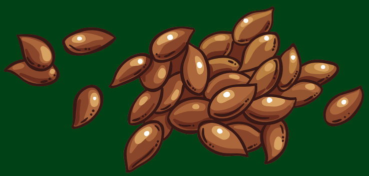

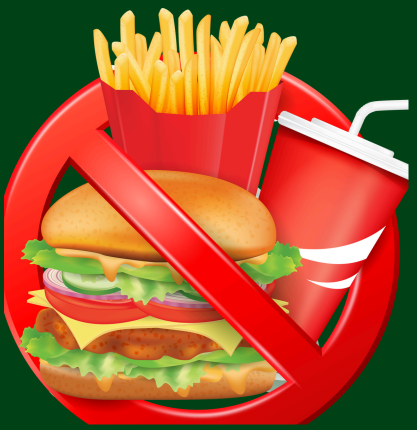

Makanan siap saji dan makanan yang di goreng termasuk dalam pantangan makanan yang menyebabkan penyakit jantung dan harus dibatasi jumlah konsumsi baiknya makanan yang di rebus atau di oven.

SALAM SEHAT!!!

Hasanah, D. Y., Zulkarnain, E., Arifianto, H., Sasmaya, H., Suciadi, L. P., Dewi, P. P., Soerarso, R., Nauli, S. E., Putri, V. K. P., Aditya, W., & Sarastri, Y. (2023). Pedoman Tatalaksana Gagal Jantung Edisi Tahun 2023. https://inahfcarmet.org/library/pedoman-tatalaksana-gagal-jantung-2023

Ndruru, A. K. (2020). Manajemen Diri Dengan Kualitas Hidup Pasien Gagal Jantung Kongestif Tahun 2020. Repository.Stikeselisabethmedan.Ac https://repository.stikeselisabethmedan.ac.id/wp-content/uploads/2021/11/ATASI-KRISMON.pdf

Pintaningrum, Y. ., rahmat, B. ., & ermawan R. (2019). Buku Ajar Ilmu Penyakit Jantung. PT. Percetakan Bali.

Rahmi, U., Keperawatan, F. I., Magister, P., Keperawatan, I., Keperawatan, K., & Bedah, M. (2018). Universitas Indonesia Pengaruh Discharge Planning Terstruktur Terhadap Kualitas Hidup Pasien Stroke Iskemik Di Rsud Al-Ihsan Dan Rs Al-Islam.

Qadrianti. (2021). Penggunaan Instrumen Untuk Menilai Kualitas Hidup Pasien Dengan Gagal Jantung di Asia. 6.

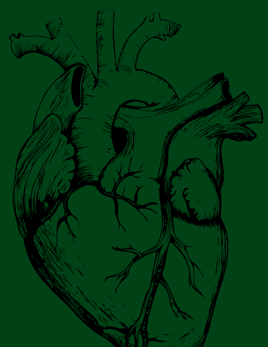

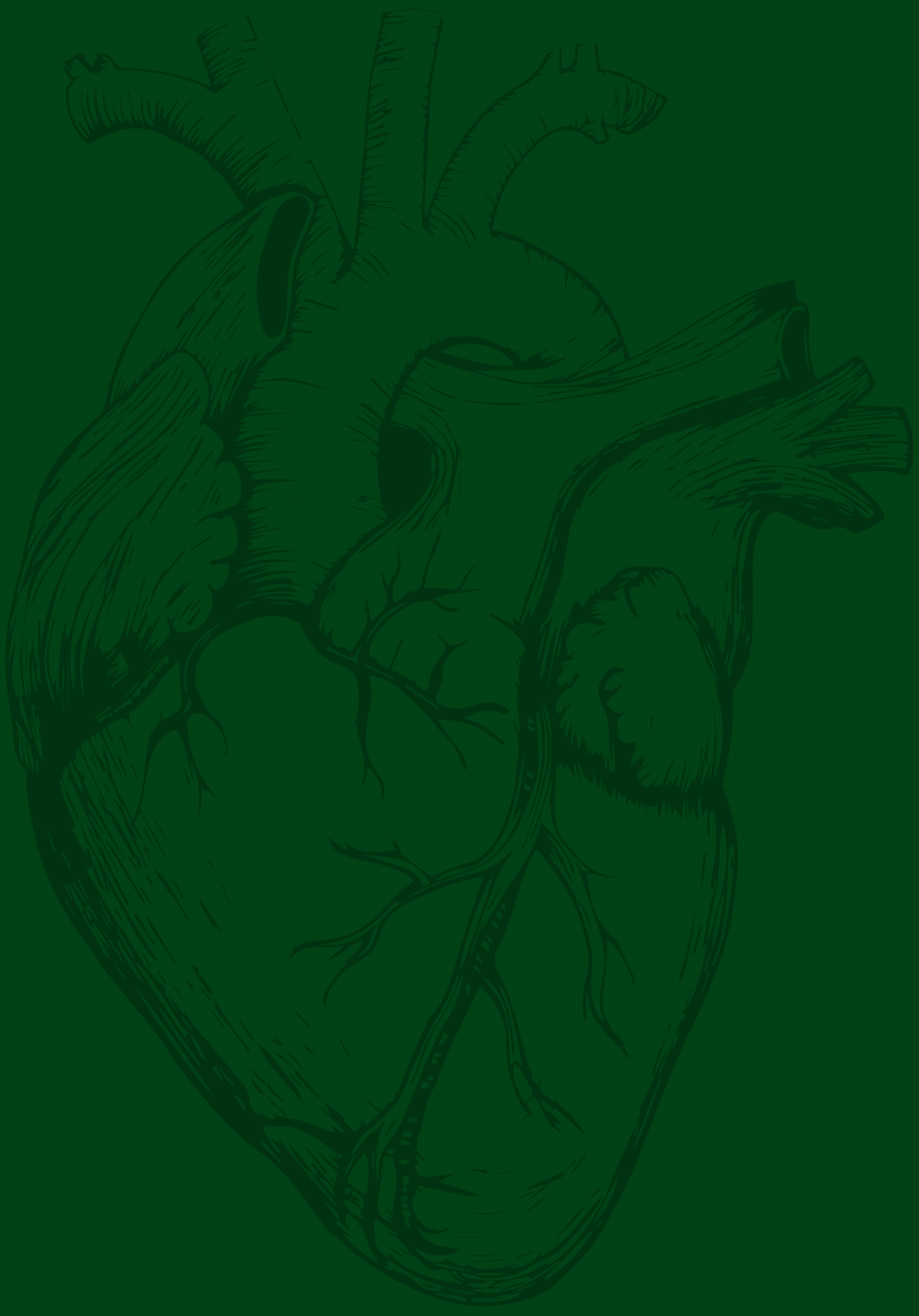

Supplement: Multimedia Appendix 1 [file cardio-v9-e75417-s001.pdf]
