# Supplementary material for: The Effect of Discharge Planning Videos and Booklets on Quality of Life Among Patients With Heart Failure: Quasi-Experimental Study
Source: JMIR Cardio. 2025 Sep 5;9:e75417. doi: 10.2196/75417 (PMC12413017; doi:10.2196/75417)
Supplement: Multimedia Appendix 2 [file cardio-v9-e75417-s002.pdf]

The Minnesota Living With Heart Failure (MLHFQ) Questionnaire
(Indonesian Version)

Pertanyaan dibawah ini mengenai seberapa besar kondisi gagal jantung yang anda derita mempengaruhi kehidupan anda dalam satu bulan terakhir ini. Berilah tanda checklist (√) pada jawaban yang menurut anda paling sesuai, untuk menentukan seberapa besar pengaruhnya dalam kehidupan anda.

Apakah penyakit gagal jantung yang anda derita mempengaruhi kehidupan yang anda jalani selama satu bulan terakhir ini, melalui hal-hal berikut ini:

No	Pertanyaan	Tidak pernah	Jarang	Sering	Selalu
1	Menyebabkan bengkak pada kaki dan pergelangan tangan				
2	Menyebabkan anda harus duduk atau tidur sepanjang hari				
3	Menyebabkan anda kesulitan saat berjalan dan naik tangga				
4	Menyebabkan anda sulit melakukan pekerjaan di sekitar rumah atau di halaman				
5	Menyebabkan anda sulit mengunjungi tempat lain di luar rumah				
6	Menyebabkan anda sulit tidur pada malam hari				
7	Menyebabkan anda kesulitan untuk melakukan kegiatan secara bersama sama dengan teman atau keluarga				
8	Menyebabkan anda kesulitan melakukan pekerjaan yang anda tekuni sebagai suatu sumber pendapatan				
9	Menyebabkan anda kesulitan melakukan rekreasi, olahraga atau hobi				
10	Membuat anda membatasi makan makanan yang disukai				
11	Membuat anda mengalami sesak nafas				
12	Membuat anda merasa cepat lelah dan kurang bertenaga				
13	Membuat anda harus di rawat di rumah sakit				
14	Membuat anda harus mengeluarkan uang untuk biaya perawatan dan pengobatan				
15	Menyebabkan anda mengalami efek samping dari pengobatan				
16	Membuat anda merasa menjadi beban bagi keluarga maupun teman				
17	Menyebabkan anda merasa tidak mampu mengendalikan diri dalam kehidupan sehari-hari				
18	Menimbulkan rasa kekhawatiran				
19	Menyebabkan anda sulit berkonsentrasi atau mengingat sesuatu				

The Minnesota Living With Heart Failure (MLHFQ) Questionnaire
(English Version)

The following questions are about how much your heart failure condition has affected your life over the past month. Please place a check mark (√) next to the answer that best reflects your experience, to indicate the extent of its impact on your life.

Has the heart failure you are experiencing affected your life over the past month in the following ways:

No	Question	Never	Rare	Seldom	Always
1	Causing swelling in your ankles or legs				
2	Making you sit or lie down to rest during the day				
3	Making walking or climbing stairs difficult				
4	Making it difficult to do household or yard work				
5	Making it difficult to go places away from home				
6	Making it difficult to sleep well at night				
7	Making it difficult to interact with or do things with your family or friends				
8	Making it difficult to work for a living				
9	Making it difficult to enjoy recreational activities, sports, or hobbies				
10	Making you eat less of the foods you like				
11	Making you feel short of breath				
12	Making you feel tired, fatigued, or low on energy				
13	Making you stay in a hospital				
14	Costing you money for medical care				
15	Causing side effects from treatments				
16	Making you feel like a burden to your family or friends				
17	Making you feel a loss of control over your life				
18	Making you worry				
19	Making it difficult to concentrate or remember things				
