# Supplementary material for: The Effect of Discharge Planning Videos and Booklets on Quality of Life Among Patients With Heart Failure: Quasi-Experimental Study
Source: JMIR Cardio. 2025 Sep 5;9:e75417. doi: 10.2196/75417 (PMC12413017; doi:10.2196/75417)
Supplement: Multimedia Appendix 3 [file cardio-v9-e75417-s003.docx]

Supplementary file 2. Normality test

| **Group** | | **Statistic** | **df** | **p-value^a^** |
| --- | --- | --- | --- | --- |
| Pretest | Intervention | 0.927 | 21 | 0.119* |
|  | Control | 0.922 | 21 | 0.094* |
| Posttest | Intervention | 0.866 | 21 | 0.080* |
|  | Control | 0.955 | 21 | 0.428* |

a = Saphiro-Wilk test; * = > 0.05
